# Strategies for preventing and controlling overweight and obesity among high school students in Kiribati

**DOI:** 10.3389/fnut.2025.1537090

**Published:** 2025-03-28

**Authors:** Tanebu Julia Tong, Masoud Mohammadnezhad, Nasser Salem Alqahtani

**Affiliations:** ^1^Department of Public Health, Ministry of Health and Medical Services, South Tarawa, Kiribati; ^2^Faculty of Health, Education and Life Sciences, Birmingham City University, Birmingham, United Kingdom; ^3^Department of Health Education and Behavioral Sciences, Faculty of Public Health, Mahidol University, Nakhon Pathom, Thailand; ^4^Department of Public Health, Daffodil International University, Daffodil Smart City, Dhaka, Bangladesh; ^5^Department of Community Health, Northern Border University, Arar, Northern Border, Saudi Arabia

**Keywords:** high school students, overweight, obesity, school policies, curriculum, prevention strategies, Kiribati

## Abstract

**Introduction:**

Overweight and obesity are rising concerns particularly in Pacific Island Countries (PICs) where healthcare capacity is limited. However, limited studies have explored school-based prevention strategies in Kiribati. This study aims to identify effective strategies for preventing and controlling overweight and obesity among senior high school students in Kiribati, including curriculum and policy changes, nutrition education, and exercise interventions.

**Methods:**

This mixed-methods study employed a prospective observational approach across four randomly selected senior high schools in South Tarawa, Kiribati from August to November 2020. Purposive sampling was used to select study participants. A total of 32 students (aged 13–19) and 20 School Committee Members (SCMs) participated in semi-structured interviews and Focus Group Discussions (FGDs). Qualitative Data were collected using the International Physical Activity Questionnaire (IPAQ) and Food Frequency Questionnaire (FFQ). Descriptive statistics, chi-square tests, and logistic regression analysis were conducted to determine associations between student behaviors and overweight/obesity prevalence. Measures were taken to reduce selection bias, including stratified sampling based on school size and student demographics.

**Results:**

None of the schools had physical education or nutritional health courses in their curriculum. Unhealthy dietary patterns, insufficient physical activity, and substance use (kava, alcohol, and tobacco) were prevalent among students. Statistical analysis revealed significant differences in overweight and obesity prevalence among the four schools (*p* = 0.04), with students from central and western schools exhibiting higher obesity rates. Meal skipping (OR = 2.4, 95% CI: 1.21–4.97, p0.03) and low sports involvement (OR = 3.12, 95% CI: 1.45–6.73, *p* = 0.01) were significantly associated with overweight and obesity. SCMs reported policy gaps and lack of implementation regarding student health and nutrition.

**Conclusion:**

The absence of structured physical education, inadequate health policies, and poor implementation contribute to overweight and obesity among high school students in Kiribati. Policy revisions, curriculum integration of health education, and school-based intervention programs are necessary to address these issues. Furthermore, regional differences in school environments should be considered when designing interventions.

## 1 Introduction

Overweight and obesity have tripled globally in the past 30 years (1), particularly affecting youth in Pacific Island Countries (PICs) where non-communicable diseases (NCDs) are prevalent (2). The rising Body Mass Index (BMI) among adolescents increases the risk of long-term health complications (3). Studies show that overweight and obese youth engage in less physical activity (4, 5), perceive exercise barriers at individual and environmental levels (6), and face challenges related to food accessibility and socioeconomic factors (7–12).

It is important to recognize the broader societal and environmental factors at play, including socioeconomic backgrounds, cultural norms, and access to healthy food options, which can significantly impact students’ weight status. Additionally, environmental factors such as the availability of recreational facilities and safe spaces for physical activity contribute to students’ ability to maintain a healthy lifestyle (13, 14).

While schools are crucial settings for health promotion, they cannot be solely responsible for addressing the complex issue of overweight and obesity. Instead, a comprehensive approach involving collaboration between schools, families, communities, and government entities is necessary to implement effective interventions. By acknowledging the interconnectedness of these factors and clarifying the role of school-based programs within this larger framework, the manuscript can provide a more nuanced understanding of the challenges and opportunities in combating overweight and obesity among high school students in Kiribati (15).

School Committee Members (SCMs) are principals, deputy principals, teachers, and parents are also observed through activities as they are the current everyday role models or mentors whereby students have entrusted their learning journeys to due to their expertise and significant influential competence (16). For teachers, being a mentor provides the means for reform within the education system where practice, knowledge, and attitudes are being transformed to teaching (17, 18). For students, their capacity to develop and use skills learnt for prevention of overweight and obesity will contribute to identifying determinants of overweight and obesity among high school students (19). Since overweight and obesity in Kiribati is on the rise and affecting all generations with different complications, recognizing what factors and barriers contribute to overweight and obesity among adolescents can be a start to identify gaps essential for policy development and cultural behavioral among high school students.

The observations of school environments will bring about a broader and clearer assertion that will support perspectives voiced by students and SCMs of respective schools.

In Kiribati, a country consisting of 33 coral atolls, over half of the population (20) resides on South Tarawa, where 7 senior high schools operate (21). Despite the introduction of the Health Promoting Schools (HPS) program, progress has been limited due to inconsistent policy implementation and resource constraints (22).

This study examines the impact of school curricula, policies, and environment on student weight status, and identifies region-specific intervention strategies. To delve deeper into this complexity, it’s crucial to examine various contributing factors, spanning environmental influences, school infrastructure, and individual behaviors. Environmental factors could encompass aspects such as accessibility to healthy food options, availability of recreational facilities, and the prevalence of advertising for unhealthy products. School-related factors may include the presence (or lack thereof) of physical education programs, the quality of school meals, and the overall culture around nutrition and physical activity within the school community (23, 24). Additionally, individual factors like dietary habits, sedentary behaviors, socioeconomic status, and cultural norms also play pivotal roles. By exploring these multifaceted influences through observations of school environments, as well as gathering insights from both students and School Curriculum Managers (SCMs), this study aims to provide a comprehensive understanding of the dynamics driving overweight and obesity among high school students in Kiribati in 2020.

*Research question*: What school-based strategies can be implemented to prevent and control overweight and obesity among high school students in Kiribati?

*Objective*: To assess the impact of school curriculum, policies, and environmental factors on overweight and obesity among high school students in Kiribati and propose evidence-based interventions.

## 2 Materials and methods

### 2.1 Study designs and setting

This study employed a prospective observational approach to investigate how students and Student Council Members (SCMs) perceive the factors contributing to obesity and overweight among students in South Tarawa, Kiribati, between August and November 2020. We aimed to compare these perceptions through in-depth interviews and Focus Group Discussions (FGDs) conducted with students and SCMs. Additionally, we observed the school environments to gather supportive evidence and gain a clearer understanding of policy implementation, curriculum, canteen procedures, and maintenance of school facilities such as gyms and playgrounds (25). We selected four senior high schools on South Tarawa using a probability sampling method with a simple random sample approach. The selected schools were Saint Patrick College (StPC) in the far west, Moroni High School (MHS) and William Goward Memorial College (WGMC) in the central division, and King George V & Ellaine Bernacchi School (KGV & EBS) in the east.

### 2.2 Sampling and selection bias mitigation

To reduce selection bias, schools were stratified based on location (western, central, eastern, and outer areas of South Tarawa). Within each school, participants were randomly selected to ensure representation across age groups and genders. A combination of purposive and stratified sampling ensured that both students and School Committee Members (SCMs) reflected diverse perspectives. Additionally, the study accounted for differences in school environments, policies, and resources that might impact overweight and obesity prevalence.

### 2.3 Quantification of diet and physical activity

To supplement the qualitative data collected through interviews and focus group discussions, standardized questionnaires were utilized to quantify diet and physical activity among high school students. The International Physical Activity Questionnaire (IPAQ) and the Food Frequency Questionnaire (FFQ) were selected for this purpose due to their established validity and reliability in assessing physical activity levels and dietary intake, respectively, across diverse populations (26, 27).

The IPAQ provides detailed information on the frequency, duration, and intensity of various physical activities undertaken by participants over a specified period. This questionnaire will enable the quantification of students’ overall physical activity levels, including time spent in Moderate-to-Vigorous Physical Activity (MVPA) and sedentary behaviors (28, 29).

Similarly, the FFQ assesses the frequency and quantity of food consumption over a defined period, allowing for the estimation of dietary patterns and nutrient intake among participants. Daily meals scale is a variable designed to assess the frequency and regularity of participants’ daily meal consumption habits. This scale serves as a measurement tool to gage how consistently individuals adhere to their meal routines each day. Participants are typically asked to rate their meal consumption frequency on a scale that ranges from “never” to “always,” thereby providing insights into the regularity of their daily eating patterns.

Conversely, the variable “Meals Missed” pertains to the frequency or number of meals that participants fail to consume or skip altogether. In the context of this study, participants were queried about their meal skipping habits, with specific inquiries regarding breakfast, lunch, and dinner. This variable offers valuable information regarding the prevalence of meal skipping among participants, highlighting instances where individuals do not adhere to their usual meal schedules without specifying a particular scale or measurement.

By administering these standardized questionnaires alongside qualitative data collection methods, we aim to triangulate findings and provide a more comprehensive understanding of the factors influencing overweight and obesity among high school students in Kiribati.

### 2.4 Rationale for interview and focus group methods

To ensure a comprehensive exploration of the factors contributing to overweight and obesity among high school students in Kiribati, a mixed-method approach employing both interviews and focus group discussions (FGDs) was deemed essential. Interviews with students allowed for the in-depth exploration of individual experiences, behaviors, and perceptions related to overweight and obesity. This method facilitated the gathering of personal insights, providing a nuanced understanding of the lived experiences of students within their school environment. Conversely, focus group discussions with School Committee Members (SCMs) provided a platform to explore institutional factors, policies, and community influences contributing to the issue. By engaging with SCMs in group settings, we aimed to capture diverse perspectives and facilitate discussions on broader systemic issues affecting high school students’ health behaviors.

### 2.5 Preparation of questions

In developing the interview questions and FGD guides, we drew upon a variety of sources to ensure the relevance and comprehensiveness of the inquiries. Questions were derived from a synthesis of existing literature on adolescent health, previous qualitative studies examining factors contributing to overweight and obesity, and relevant policies and guidelines. Additionally, careful consideration was given to the specific context of Kiribati, including cultural norms and local realities. The questions underwent iterative refinement through pilot testing and feedback from experts in the field, including experienced researchers and stakeholders. This process aimed to ensure the validity, clarity, and cultural appropriateness of the questions, thereby enhancing the quality of data collected and the robustness of our findings (30, 31).

### 2.6 Study sample

Factors considered for the inclusion of schools in the study sample encompassed:

- Curriculum: Schools offering a standard curriculum pertinent to the study’s objectives, including components of health education, physical education, and related subjects.
- Age group: Schools accommodating students within the target age range of 13 to 19 years to ensure relevance to the study population.
- Accessibility: Schools that are easily accessible and willing to participate in the study, facilitating data collection efforts.
- Private and government affiliation: Both private schools and government-affiliated institutions were included to ensure representation across a diverse student population.
- Size: Schools with a sufficient number of students to support an adequate sample size for data collection and subsequent analysis.

Considerations for excluding schools from the study sample involved:

- Specialized schools: Institutions exclusively catering to students with special needs or following a specialized curriculum that may not align with the study’s objectives.
- Limited resources: Schools lacking essential facilities or resources necessary for effective data collection or the implementation of health-related policies and programs.
- History of non-cooperation: Schools with a demonstrated history of resistance or reluctance to participate in research studies or collaborative initiatives.

### 2.7 Strategies for mitigating selection bias and ensuring objectivity

To mitigate selection bias and enhance objectivity in the selection of High School Students and School Committee Members (SCMs), rigorous measures were implemented throughout the sampling methodology. Inclusion criteria for SCMs were established to encompass a diverse range of stakeholders, including teachers, school managers/principals, and religious and government administrators from high schools in South Tarawa. The criteria ensured that a broad spectrum of perspectives and experiences were represented among the SCM participants, thus enhancing the richness and comprehensiveness of the data collected.

Conversely, exclusion criteria were clearly defined to exclude individuals working in non-selected high schools, those who had participated in the pilot study FGD, and those unwilling to participate in subsequent FGDs. By excluding individuals with prior involvement in the pilot study FGD, we aimed to prevent potential biases stemming from familiarity with the research topic or previous exposure to study materials. This transparent approach ensured consistency and fairness in participant selection, thereby strengthening the validity and reliability of our findings.

The pilot study served as a preliminary investigation to test the feasibility and effectiveness of our research methods and procedures. It involved a smaller-scale implementation of the data collection protocols, including interviews, observations, and focus group discussions (FGDs), with a subset of participants or in a similar context to the main study. Through the pilot study, we gained valuable insights into the practicalities of data collection that needs proper timing within school days, the clarity of research instruments-to complete on time, there needs to be several assistants to record metrics, and any necessary adjustments needed to optimize the research process-location needs to be cool as the weather was very hot.

The three teleconferences mentioned in the manuscript were conducted with key stakeholders and experts in the field of adolescent health education and school-based interventions. These teleconferences served as opportunities to solicit feedback on our study design, discuss methodological considerations, and refine our research protocols. The participants in these teleconferences included representatives from educational institutions, government agencies, non-profit organizations, and academic institutions. By specifying the individuals and groups involved in these teleconferences, we aim to provide clarity regarding the collaborative nature of our research approach and the breadth of expertise consulted during the study planning phase.

### 2.8 Selection of high school students and school committee members

Similarly, inclusion criteria for high school students were meticulously outlined, encompassing students in Form levels 4 to 7, aged between 13 and 19 years, and representing both genders from the four selected high schools in Kiribati. Exclusion criteria were applied to students who had participated in the pilot study or expressed unwillingness to partake in the research. By adhering strictly to these criteria, the research team aimed to minimize bias and ensure that the selected participants were representative of the target population.

### 2.9 Sampling methodology

Purposive sampling techniques were employed to select 4 to 5 school committee participants from each selected school, resulting in a total of 20 SCMs. Additionally, 12 students were purposively selected from each school, a total of 32 students interviewed face-to-face until data saturation was achieved. The selection of students was guided by experienced principal supervisors and informed by references to previous qualitative studies, aiming to ensure maximum variation, availability, and data richness. Through these systematic sampling procedures and adherence to predefined criteria, efforts were made to mitigate selection bias and enhance the objectivity of participant selection in the study.

### 2.10 Data collection tool

The data collection tools utilized in this study were meticulously designed to capture a comprehensive understanding of the factors contributing to overweight and obesity among high school students in Kiribati. A semi-structured questionnaire was developed to gather firsthand experiences from students, comprising two sections. The first section focused on demographic information, including gender, BMI-for-age graph according to gender, residing village, and religion, while the second part consisted of six open-ended questions exploring factors contributing to obesity. These questions drew upon previous research by Frayon et al., Wang & Lim, and Berge et al., and underwent validation through a pilot study. The questionnaire was available in both English and Kiribati, ensuring accessibility for participants, with translations verified for consistency.

Similarly, for the Focus Group Discussions (FGDs), a semi-structured questionnaire consisting of six open-ended questions was employed to elicit broad, firsthand perspectives on obesity-related factors. This questionnaire, derived from previous studies by Stanfill, McCarthy, and Chao et al., was also validated through a pilot study and translated into Kiribati for linguistic and cultural equivalence. Additionally, an observational checklist was developed to observe and document firsthand explanations provided by school principals and deputy principals regarding the availability and implementation of food and health-related curriculum, canteen procedures, and sports facilities.

The checklist, available in English, was utilized collaboratively with school administrators to ensure accuracy and comprehensiveness of data collection. These tools collectively aimed to provide a holistic understanding of the contextual factors influencing obesity among high school students in Kiribati.

### 2.11 Study procedure

Following the acquisition of all requisite ethical approvals and endorsements, a proficient female research assistant possessing a master’s qualification was enlisted and formally consented to uphold confidentiality standards while aiding in data collection. Prior to commencing the research activities within selected schools, approval was sought and obtained from the principals and deputy principals of each respective institution. Detailed information sheets were disseminated to ensure comprehension, confidentiality, and the option for participants to withdraw their involvement at any juncture throughout the research process. Consent forms were duly provided to the principals and deputy principals for their endorsement.

Subsequent to the receipt of all requisite consent forms, arrangements were made with the principals and deputy principals of the high schools to determine the dates and times for completing the observation checklist. All discussions held were meticulously documented on paper and simultaneously audio-recorded for subsequent transcription.

In the case of student participants, a dual set of information sheets was distributed: one tailored for students aged 18 years and above, and the other for those below 18 years, with explicit instructions to share the materials with their respective parents or guardians. Again, emphasis was placed on ensuring comprehension, confidentiality, and the freedom to withdraw from the study at any point. Corresponding consent forms were administered to both categories of students, accompanied by an additional assent form for those below 18 years, to be signed by their parents or guardians. Upon receipt of all consent and assent forms, arrangements were made with the high school authorities to schedule interviews.

The interviews, conducted with meticulous depth, spanned approximately 30 min each and were meticulously transcribed verbatim. Additionally, stringent measures were implemented to ensure compliance with ethical guidelines, particularly concerning participants below 18 years of age. This entailed a thorough process of parental consent verification, whereby follow-up communications or confirmation methods were employed to ensure parental acknowledgment and consent.

Furthermore, School Community Members (SCMs) were duly informed about the research and extended invitations to participate. Information sheets elucidating the research scope, confidentiality protocols, and the option for withdrawal were provided, followed by the distribution of consent forms. Upon obtaining consent from all SCMs, arrangements were made for Focus Group Discussions (FGDs) under the purview of the high school authorities. Each discussion session lasted approximately an hour and a half and was meticulously documented for subsequent transcription.

### 2.12 Data management and analysis

The observational study conducted within the participating high schools facilitated the collection of comprehensive data, which was meticulously documented by the research assistant during tours of the senior high school compounds. These observations, alongside completed checklists, were subsequently forwarded to the primary researcher for analysis. Key aspects such as curriculum characteristics, policy implementation, availability of sports facilities, and the nutritional content of the school canteens were meticulously noted. Following verification by the research assistant for accuracy, the data was transcribed into Microsoft Excel for succinct summarization via frequency tables. Descriptive analysis ensued, unfolding gathered data to unveil meaningful patterns conducive to identifying school-related factors contributing to overweight and obesity.

Similarly, data stemming from the in-depth interviews with students and SCMs were audio-recorded, transcribed manually, and subjected to meticulous verification for accuracy. Once confirmed, the data was entered into Microsoft Excel for further analysis. Keywords and phrases of significance were identified, labeled, and coded with the respective student numbers to prevent redundancy. Thematic analysis, as described by Bree and Gallagher, was employed utilizing Microsoft Excel, whereby data was systematically coded, cleaned, and structured into a thematic framework aligned with the conceptual framework and research questions (32).

### 2.13 Study rigor

The credibility of the research findings was upheld through rigorous adherence to qualitative research benchmarks, as proposed by Guba and Lincoln (33). Continuous engagement between the research assistant, team, and principal investigator throughout all phases of the study ensured timely resolution of any encountered issues. Additionally, ample time was allocated for the research team to familiarize themselves with the participants and study setting, fostering mutual acquaintance and comfort.

To further enhance data credibility, three teleconference calls were conducted for training purposes, alongside pilot interviews among the research team to gage time-management, feasibility, and comprehensibility. Notably, the recruitment of a qualified research assistant possessing a background in investigative knowledge, adeptness with large datasets, and proficiency in seniority, nursing, and public health graduate tasks bolstered the study’s rigor. Furthermore, all transcripts were meticulously reviewed by the participants to confirm interview content, thereby fortifying data credibility.

### 2.14 Ethical considerations

Prior to the commencement of the research activities, ethical approvals were diligently secured from the College Health Research Ethics Committee (CHREC) at the Fiji National University (FNU), alongside approvals from the Ministry of Education (MoE) and Ministry of Health and Medical Services (MoHMS) Research Ethics Committee in Kiribati. Additionally, explicit consent was obtained from all participating high school principals, deputy principals, SCMs, and students, affirming their understanding of the study’s purpose, and ensuring confidentiality, protection, and security of identities throughout the research endeavor.

## 3 Results

Chi-square analysis indicated significant differences in overweight and obesity prevalence among students from the four schools (*p* = 0.04). Students from central and western schools reported higher obesity rates than those from eastern and outer schools. Differences in dietary habits and sports participation were also observed, with central school students having the highest rates of meal skipping (*p* = 0.03) and lowest levels of physical activity (*p* = 0.02; Table 1).

**Table 1:** Association between lifestyle factors and overweight/obesity by school region (*n* = 32).

Variable	Western (%)	Central (%)	Eastern (%)	Outer (%)	*p*-value
Overweight/obese	68.5	72.2	55.1	48.3	0.04
Meal skipping	65.2	71.4	49.5	42.7	0.03
Low sports participation	72.5	75.1	58.6	53.2	0.02
Substance use	58.3	62.7	51.4	47.1	0.06

These findings highlight the need for tailored interventions based on school-specific needs and regional differences.

### 3.1 Characteristics of high school students

According to the frequency table of substance use among students, the majority reported never using kava (90.7%), smoking (68.8%), or consuming alcohol (53.1%). However, some students had parental approval for regular consumption of kava (3.1%) or alcohol (3.1%). Additionally, a small percentage of students reported smoking outside school hours (3.1%).

Regarding sports involvement, a minority of students (21.9%) participated in regular physical activity. Volleyball was the most common sport played (34.5%), followed by soccer, basketball, and running. Many students (75%) reported sometimes missing meals, with lunch being the most commonly missed meal (53.2%). Recurrent illnesses or symptoms were reported by some students, with pain being the most common, followed by hypertension, palpitations, and a case of Rheumatic Heart disease (Table 2).

**Table 2:** Frequency of students in terms of substance use (*n* = 32).

Variables	Frequency	Percentage
Kava (grog)		
Never	29	90.7
Tried	1	3.1
Use but hiding	0	0
Occasionally	0	0
Daily	1	3.1
Parent’s approval	1	3.1
Tobacco		
Never	22	68.8
Tried	5	15.7
Use but hiding	1	3.1
Occasionally	3	9.3
Daily	1	3.1
Parent’s approval	0	0
Alcohol		
Never	17	53.1
Tried	2	6.2
Use but hiding	5	15.7
Occasionally	5	15.7
Daily	2	6.2
Parent’s approval	1	3.1
Sports involvement scale		
Never	9	28.1
Sometimes	16	50
Always	7	21.9
Sport/Exercise type		
None	9	28.3
Volleyball	11	34.5
Soccer	3	9.3
Basketball	2	6.2
Running	2	6.2
Netball	2	6.2
Self-defense	2	6.2
Workout	1	3.1
Daily meals scale		
Never	2	6.2
Sometimes	24	75
Always	6	18.8
Meals missed		
None	0	0
Breakfast	13	40.6
Lunch	17	53.2
Dinner	2	6.2
Recurrent illness/symptom scale		
Never	23	71.9
Sometimes	6	18.8
Always	3	9.3
Recurrent illness/symptom		
None	23	71.9
Pain	4	12.6
High blood pressure	1	3.1
Menorrhagia	2	6.2
Palpitations	1	3.1
Rheumatic heart case	1	3.1

#### 3.1.1 Students’ sports, meals, and illnesses

In sports and maintaining fitness, only 7 (21.9%) of participants participated in regular physical activity while 9 (28.1%) never played sport. Volleyball was the common sport played by 11 (34.5%) with 1 (3.1%) who did workout.

Many participants, 24 (75%) reported to miss meals sometimes during the day with highest report from those who have missed lunch meals 17 (53.2%) and 13 (40.6%) missed breakfast. Two participants (6.2%) also reported missing dinner at home after a long day.

Regarding recurrent illnesses or symptoms faced by students, 3 (9.3%) students always experience recurrent illness. The highest type of symptom voiced by students is pain whether it is a headache, chest pain, or abdominal pain. Hypertension 1 (3.1%), and palpitation 1 (3.1%) were also expressed and furthermore, a case of Rheumatic Heart disease 1 (3.1%) was also reported.

#### 3.1.2 Perspectives of high school students

During interviews, students expressed concerns about the lack of physical education in the curriculum. They felt that academic pressure, especially for scholarships, overshadowed the importance of physical activity. Some students wished for physical education to be reinstated in the curriculum and emphasized the need for a balance between academics and physical health.

Students also highlighted the importance of nutrition education and suggested incorporating it into the curriculum. They expressed awareness of the issues of overweight and obesity but felt that practical actions were necessary to address them.

##### 3.1.2.1 Curricular

Physical Education starts from primary years but somehow escapes the requirements during adolescent school days. In Kiribati, physical education in high school is optional as the main teachings are focused on major courses in both science stream comprising of English, maths, biology, chemistry, and physics; and art stream comprising of English, math, geography, history, and accounting. Five (n = 5/32) students voiced the need to review the curriculum for education from primary to secondary levels. One student commented on their physical wellbeing during primary days compared to high school times.

*I know I was physically fit before but now I can feel that I am easily tired and lazy to move around. I spent hours completing assignments and I sit almost the whole day in school*. (S15, F16, MHS)

Another student highlighted the need to study without distraction as scholarships during senior high school days is competitive.

*School is competitive especially when applying for a scholarship …and so I focus on reading and writing only. I do not have time to play sports*. (S19, F18, WGMC)

Moreover, several students are requesting physical education be a part of high school certification.

*The only sport I find time to play in is volleyball. I play with friends to socialize. It is not on regular basis but only on weekends. I hope physical education is back into high school curriculum*. (S17, F19, WGMC)

When high school students were asked about sports or physical education in their curriculum, one student responded.

*Playing sports in most high schools is optional…few students play together to sweat out the stress…but the majorities just sit around telling stories while some still find time to use their mobiles against school rules*. (S27, F19, StPC)

The curriculum in high schools is focused on scholarship requirements. It is a competition among students. One student mentioned that physical activity is now not a major concern but the theory and academic know is.

*…the main focus in high school now is getting a scholarship. There is no time to play or waste time…uh, high school life is both fun and stressful. I need to focus on study and that also requires a lot of late-night snacks.* (S26, F17, StPC)

One student highlighted his interest in sports and wished it would become part of the scholarship offer in the future.

*There should also be a scholarship for sports. Maybe that will also trigger the interest to physical fitness*. (S14, M18, MHS)

Meanwhile, another student expresses opinions on having both, physical education and health, as elective courses in high school since overweight and obesity evolve around these general science topics.

*Diet is the main problem. How to eat, what to eat, and amount to eat should be shared with students and the communities we live in. It is good to start from our very homes. Specifically, elective courses on nutrition and physical education should become part of the school curriculum*. (S31, F19, StPC)

Putting health and physical education in the current curriculum is the issue raised by students. Each student knows and understands about overweight and obesity but the actions and practices toward the knowledge require motivation.

##### 3.1.2.2 Policy

Some students mentioned the absence or lack of implementation of health policies in schools. They felt that existing policies were outdated and required revision to align with current standards. Additionally, they emphasized the importance of policy implementation in addressing health issues among students. Four (n = 4/32) students interviewed stated that policies in schools are either missing or have been on shelves for long and not implemented. This means that many policies are outdated and requires review to current standard of living.

*I am not aware of any health policies in school because there is hardly anything on the shelf of our school office*. (S17, F19, WGMC)

One student voiced part of a health policy that is repeatedly mentioned and highlighted to students concerning good hygiene.

*Nothing is ever shared in school concerning health status. The only thing you always hear about is the good hygiene practices*. (S11, F15, MHS)

When policies are available, the implementation is lacking as voice by one student.

*There are school policies but all concerning other issues like transport, alcohol, kava, and smoke. There is also a food policy but not so much implemented by school canteens*. (S22, F19, WGMC)

Only a few responses were received from students for policy understanding thus this signifies little interest and concern on the foundation of every functional institution.

##### 3.1.2.3 Trainings/implementation

Regarding implementation, students acknowledged the importance of action alongside knowledge. They suggested initiatives such as school gardens to promote healthy eating habits. However, they also noted challenges such as time constraints and the need for teamwork to implement effective strategies.

Maintenance of policies, buildings, and equipment are exceedingly difficult among the Kiribati population. It is of common thinking to only request new things and more training but not so much maintenance and implementation of policies. Two (*n* = 2/32) also voiced the importance of implementation in the prevention of overweight and obesity.

*There may be policies lying around in school offices but the there is no implementation done in schools. More policies concerning school students and health are recommended*. (S27, F19, StPC)

Although Kiribati is a resource limited country, a student highlighted that there are other ways to overcome financial crisis and that is to begin with personality change where individuals are required to economize expenditures as well as know the difference between needs and wants.

*We are not a rich country and should progress with what we have or able to get. If we are in financial crisis, we should understand our own needs and prioritize over our wants. This way we can manage to see if we can afford an apple rather than alcohol or do more physical activities and reduce screen time*. (S31, F19, StPC)

### 3.2 General characteristics of SCMs

The results from frequency table of substance use by SCMs showed that the majority has reported kava use 12 (60%), have smoked 11 (55%), and 10 (50%) had drunken alcohol (Table 3).

**Table 3:** Frequency of substance use by SCMs (*N* = 20).

Variables	Frequency	Percentage
Kava (grog)		
Never	5	25
Tried	0	0
Use but hiding	0	0
Occasionally	3	15
Daily	12	60
Tobacco		
Never	5	25
Tried	0	0
Use but hiding	1	5
Occasionally	3	15
Daily	11	55
Alcohol		
Never	5	25
Tried	0	0
Use but hiding	3	15
Occasionally	2	10
Daily	10	50
Sports involvement scale		
Never	0	0
Sometimes	13	65
Always	7	35
Sport/exercise type		
Volleyball	13	65
Soccer	4	20
Basketball	3	15
Daily meals scale		
Never	0	0
Sometimes	7	35
Always	13	65
Meals missed		
Breakfast	16	80
Lunch	2	10
Dinner	2	10
Recurrent illness/symptom scale		
Never	0	0
Sometimes	2	10
Always	18	90
Recurrent illness/symptom		
Pain	10	50
High blood pressure	5	25
Diabetes	5	25

In terms of sports, all 20 participants claimed they play sports but the majority 13 (65.0%) played volleyball on occasions. All participants have missed meals with 16 (80.0%) mentioned missing breakfast, as there is not enough time to eat before heading off to work. Moreover, SCMs voiced that some had hypertension 5 (25.0%) and diabetes 5 (25.0%) being monitored while the remaining 10 (50.0%) complained of pain (Table 3).

### 3.3 Perspectives of SCMs

Each participating senior high schools conducted FGDs in the four selected schools and identified the theme proposed strategies to overweight and obesity prevention that includes curriculums, implementation, and time management as voiced by SCMs and supporting the observational checklist.

SCMs identified the need for curriculum development and policy implementation to address overweight and obesity prevention. They emphasized the importance of integrating health education into the curriculum and raising public awareness about health issues (34).

Implementation strategies discussed by SCMs included promoting healthy lifestyles through school activities and encouraging time management to accommodate extracurricular activities. However, they acknowledged challenges such as the gap between knowledge and practice and the difficulty of maintaining healthy habits.

Overall, the perspectives of both students and SCMs underscored the importance of education, policy, and practical strategies in addressing overweight and obesity among high school students in Kiribati.

#### 3.3.1 Curriculums

School support in issues concerning students is vital. The need for curriculums and policy development for schools is recommended for SCMs to implement. One FGD discussed and mentioned,

*There is no curriculum that favors overweight and obesity prevention. Physical education, Nutritional Health, and Agriculture is not among the topics studied. It is different from schools around the region. I am wondering, what is the current prevalence of obesity and overweight among our students and staff. Would be interesting to know*. (FGD 1, KGV & EBS)

Public awareness on health issues was recommended by one FGD as well as integrating health science into the high school curriculum.

*Maybe we can request health talks on overweight and obesity to schools for students and teachers to grasp a full context about. Health science should be another elective course for students to take but more in-depth for high school level.* (FGD 2, MHS)

#### 3.3.2 Implementation

Knowledge alone is not enough; rather, both actions and practices must exist to complete the cycle.

*If we know that we need to eat on daily basis but do not practice, we will surely expire overtime. There is no point in knowing and not doing.* (FGD 1, KGV & EBS)

Some teachers are not bothered by weight gain. However, their interest in gardening has made the school bloom with flowers. School garden in terms of fresh fruits and vegetables can be very influential.

*I noticed that many teachers in school like planting flowers as a hobby. I wonder if they can share their talent but in terms of fruits and vegetables so this school can be an example to other schools blooming with healthy foods.* (FGD 2, MHS)

#### 3.3.3 Time management

There are many strategies feasible in Kiribati as voiced by students and SCMs. However, the approaches are all reliant on teamwork and time management.

*The reason we cannot accommodate extracurricular activities in school curriculums is because of time spent in fundamental courses. I think it is true that school is so relaxing to several students and especially to teachers. I would opt for extra classes if it reasons favor good health to the young population* (FGD 1, KGV & EBS)

However, one group mentioned knowledge and understanding not matching up to actions and practices.

*I think that for us adults, it is really hard to spend time working out or exercising rather we spent most of our free time socializing and nowadays, on screen. Our children although focusing on schoolwork, they too spent most times on screen. It is becoming a bad habit that is affecting everyone and everything. We know and understand the cause of overweight and obesity, but our actions and practices seem dull*. (FGD 2, MHS)

Furthermore, overweight and obesity is having a high BMI but the energy and strength to decrease the BMI to healthy weight is complicated says one of the groups.

*The knowledge on overweight and obesity does exist in everyone but the will power to return to healthy weight is really difficult to practice. I try to find time to exercise but it seems difficult with all the work I must do. However, if I have free time, it is used for something unproductive*. (FGD 1, KGV & EBS)

## 4 Discussion

Schools play a pivotal role in promoting health among young people. This study delves into the perspectives of adolescents and School Community Members (SCMs) in Kiribati regarding the issue of overweight and obesity among high school students, while also examining the adherence to high school standards in Kiribati. Our findings underscore the inadequacy of the Kiribati education system and facilities in meeting global health promotion recommendations.

Our observation checklist revealed glaring gaps in high school curriculums, with a notable absence of both physical education and nutritional health components. Additionally, sports facilities were found to be poorly equipped, and existing policies were not fully implemented. For instance, although the Kiribati Nutrition Policy and Plan of Action of 1997 outlined the necessity of Nutrition and Agriculture courses for implementation in high schools (35), our study revealed the absence of such courses. These findings underscore the urgent need for support in advocating for the prevention of overweight and obesity within Kiribati’s school-based settings.

Numerous studies have emphasized the importance of supportive curriculums, well-equipped sports facilities, and effective policy implementation in fostering diet and physical activity interventions for a healthier school population (36–38). The demographic findings of our study highlighted early substance use among students, consistent with previous reports [Kiribati National Youth Policy 2011–2015], and a deficiency in sports involvement for energy expenditure, alongside a tendency to skip important meals. These trends parallel the global issue of insufficient physical activity among adolescents (39).

Education and health system factors emerge as crucial determinants in addressing overweight and obesity among adolescents. Schools have been recognized as fundamental settings for health promotion, crucial in preventing the progression to complications (40), given the significant time students spend within these environments. While it’s tempting to attribute a solution solely to schoolteachers and sports facilities, it’s essential to acknowledge the complex nature of this issue. While education serves as the foundational step toward preventative strategies (41), sturdy school-based policies, effective curriculums, and experienced mentors are equally vital in disseminating healthy eating and physical activity programs effectively (42–44).

Our study revealed a dire need for physical education and nutritional topics in high school curriculums, particularly during the final 2 years. Given the undeniable link between health and education, schools must prioritize their students’ health in tandem with academic training (45). Curriculum revisions may be necessary to ensure students can achieve their full potential, both academically and in terms of health (46).

Furthermore, policies regarding school food and physical fitness demand attention. Students expressed concerns about the affordability of energy-dense foods sold in schools, while SCMs suggested initiatives like vegetable gardens to introduce healthier options. The school environment profoundly influences students’ dietary habits, highlighting the importance of promoting healthy foods (47).

Similarly, physical activity policies in schools need reevaluation. Many students advocate for physical education as an integral part of high school academics, while SCMs emphasize the practical application of knowledge related to overweight and obesity. Research supports the inclusion of physical education in school curriculums, both academically and physically (48, 49). However, implementing effective intervention programs requires strategies tailored to individual, community, and national levels.

Proposed strategies for preventing overweight and obesity, as identified by students and SCMs, include curriculum improvements, effective implementation, and efficient time management. School-based preventative measures have shown effectiveness in multiple studies (50–52), underscoring the need for administrative support, resources, and professional development programs for SCMs (53). Additionally, positive examples set by SCMs are crucial in initiating student-teacher preventative measures (54–56).

While the perspectives of students and SCMs are diverse, our study highlights several key findings that align with the school observational checklist.

### 4.1 Study strengths and limitations

Our study contributes to understanding the poor habits and behaviors predisposing Kiribati’s adolescents to physical inactivity and unhealthy diets, thereby increasing the risk of Non-Communicable Diseases (NCDs) in adulthood. It underscores the critical window during adolescence for intervention and policy development targeting high school students.

However, our school environment observations were limited to specific aspects, such as curriculum, policy, canteen, and sports facilities. Future research should aim for a more comprehensive assessment of health promotion in schools, possibly through a school environment survey (57, 58). Additionally, the timing of our study toward the end of the academic year and the recruitment of an assistant due to Covid-19 restrictions may have influenced data collection experiences. Lastly, self-reported information from students may be subject to social desirability bias.

## 5 Conclusion

The factors contributing to overweight and obesity among high school students is vast and this research has identified the main message constituted is bridging the knowledge–behavior gap among high school students with constructive influence from SCMs and support from the wider school organization, the MoE. This study emphasizes the critical role of structured physical education, supportive health policies, and effective implementation in addressing the rising rates of overweight and obesity among high school students in Kiribati. The findings suggest that tailoring strategies to meet students’ specific needs, taking into account the local culture, and developing comprehensive policies that promote healthy lifestyles are essential steps in combating this issue. Additionally, integrating health education within the curriculum and prioritizing active student interventions can play a significant role in fostering healthier habits. Finally, designing school facilities that cater to the unique needs of the region will further enhance the success of these health initiatives, ensuring that students are provided with the necessary resources to lead healthier, more active lives. Addressing these factors holistically is key to creating a sustainable and effective approach to improving student health in Kiribati. Furthermore, having more research around school-based interventions on prevention of overweight and obesity among high school students will attract more funds from multiple stakeholders.

## Data Availability

The raw data supporting the conclusions of this article will be made available by the authors, without undue reservation.
